# Metagenomic Frameworks for Monitoring Antibiotic Resistance in Aquatic Environments

**DOI:** 10.1289/ehp.1307009

**Published:** 2013-12-13

**Authors:** Jesse A. Port, Alison C. Cullen, James C. Wallace, Marissa N. Smith, Elaine M. Faustman

**Affiliations:** 1 Department of Environmental and Occupational Health Sciences, and; 2 The Institute for Risk Analysis and Risk Communication, School of Public Health, University of Washington, Seattle, Washington, USA; 3 Evans School of Public Affairs, University of Washington, Seattle, Washington, USA

## Abstract

Background: High-throughput genomic technologies offer new approaches for environmental health monitoring, including metagenomic surveillance of antibiotic resistance determinants (ARDs). Although natural environments serve as reservoirs for antibiotic resistance genes that can be transferred to pathogenic and human commensal bacteria, monitoring of these determinants has been infrequent and incomplete. Furthermore, surveillance efforts have not been integrated into public health decision making.

Objectives: We used a metagenomic epidemiology–based approach to develop an ARD index that quantifies antibiotic resistance potential, and we analyzed this index for common modal patterns across environmental samples. We also explored how metagenomic data such as this index could be conceptually framed within an early risk management context.

Methods: We analyzed 25 published data sets from shotgun pyrosequencing projects. The samples consisted of microbial community DNA collected from marine and freshwater environments across a gradient of human impact. We used principal component analysis to identify index patterns across samples.

Results: We observed significant differences in the overall index and index subcategory levels when comparing ecosystems more proximal versus distal to human impact. The selection of different sequence similarity thresholds strongly influenced the index measurements. Unique index subcategory modes distinguished the different metagenomes.

Conclusions: Broad-scale screening of ARD potential using this index revealed utility for framing environmental health monitoring and surveillance. This approach holds promise as a screening tool for establishing baseline ARD levels that can be used to inform and prioritize decision making regarding management of ARD sources and human exposure routes.

Citation: Port JA, Cullen AC, Wallace JC, Smith MN, Faustman EM. 2014. Metagenomic frameworks for monitoring antibiotic resistance in aquatic environments. Environ Health Perspect 122:222–228; http://dx.doi.org/10.1289/ehp.1307009

## Introduction

Advances in genomic technologies now offer novel approaches for environmental health monitoring and risk assessment. High-throughput sequencing of whole microbial communities provides global snapshots of community and functional composition, as opposed to more conventional analyses that are species and gene specific (Hugenholtz and Tyson 2008). Because these new techniques rely on culture-independent approaches, they are able to access genomic information from the vast majority of bacteria that are not culturable (Amann et al. 1995). These technologies are also less labor and laboratory intensive and can generate massive volumes of genomic data in less than a day (Glenn 2011). Shotgun metagenomics, or the direct extraction, sequencing and analysis of DNA from a community of microorganisms (Handelsman 2004), is one high-throughput approach that in tandem with next generation sequencing has potential utility for environmental public health surveillance.

Although the environmental health applications of metagenomics remain to be fully elucidated, this approach has been used to track fecal contamination in watersheds via community composition profiling (Wu et al. 2010), detect pathogens in wastewater (Ye and Zhang 2011), and identify indicators of sewage contamination (Bibby and Peccia 2013; McLellan et al. 2010). Although these techniques are thus promising, interpretation of the massive amounts of data produced poses a series of challenges for public health decision makers. Determining the significance of a given genomic signal in the context of risk, defining the levels of genomic response needed to drive a decision, and identifying the cost-benefit balance of using these methods versus more traditional approaches will be necessary to translate metagenomic data into public health decision making. Here we present a first step toward developing a decision-monitoring tool using the case study of antibiotic resistance in marine and freshwater environments.

Antibiotic resistance is a global phenomenon and is a growing source of morbidity and mortality (Bush et al. 2011). Resistance occurs when bacteria evolve under selective pressure to confer resistance to antibiotics used to treat their infection. Although the majority of antibiotic resistance investigations have been focused on pathogenic bacteria in clinical settings, antibiotic resistance and antibiotic resistance determinants (ARDs) have been shown to be widespread in environmental bacteria (Wright 2010); furthermore, many resistance genes found in pathogenic bacteria have evolved or are sourced from environmental microbial communities (Martinez 2009). ARDs refer here to the genomic factors related to the presence and dissemination of antibiotic resistance genes (ARGs), including mobile genetic elements (MGEs) such as plasmids, transposable elements (TEs), and phages as well as metal resistance genes (MRGs), which have been shown to co-select for ARGs (Wright 2007). The antibiotic resistomes of natural environments including soil, marine, freshwater, and wastewater ecosystems have revealed an abundance of ARDs (Allen et al. 2010; Davies and Davies 2010; Zhang et al. 2009). In many cases, these genes have been shown to be functionally resistant to selected antibiotics (Schmieder and Edwards 2012). The presence of resistance genes in the environment may be due to selective pressures favoring these genes, including antibiotic overuse and misuse in clinical treatment and agricultural and aquaculture applications as well as metal pollution. ARDs are ultimately disseminated into watersheds and coastal systems via sewage, animal waste, and urban/agricultural runoff, and thus form environmental reservoirs of ARDs (Davies and Davies 2010). Humans can be exposed through food, including crops, livestock, and seafood; consumption of contaminated drinking water; recreational activities such as swimming; or direct contact with organisms carrying antibiotic resistant bacteria (Wellington et al. 2013).

Monitoring for antibiotic resistance in the marine environment has been infrequent and incomplete (Allen et al. 2010) and has predominantly focused on measuring levels of antibiotics in different water matrices (Segura et al. 2009). Furthermore, environmental monitoring of antibiotic resistance has not been formalized into public health surveillance or water quality management decision frameworks, likely because of a continuing lack of data and uncertainty regarding risk and risk metrics. Instead, global surveillance efforts such as the European Antimicrobial Resistance Surveillance Network (European Centre for Disease Prevention and Control 2014) and the National Antimicrobial Resistance Monitoring System for Enteric Bacteria (Centers for Disease Control and Prevention 2014) have predominantly focused on the prevalence of antibiotic usage and antibiotic resistance isolates in clinical and public health laboratory settings (Grundmann et al. 2011). Given the global magnitude of antibiotic resistance, including the emergence of multi-drug resistance bacterial strains and increasing reports of occurrence in the environment, there is a critical need for the identification, characterization, and control of these generally uncharacterized environmental ARD reservoirs (Bush et al. 2011).

The objectives of the present study were three-fold. First, a metagenomic epidemiology-based approach was used to develop an index that quantifies the resistance potential of an environment. Metagenomic epidemiology is a multi-layered approach that considers the entire microbiotic context for environmental antibiotic resistance by characterizing simultaneously the different levels of microbiome complexity that drive antibiotic resistance including ARGs, genetic vectors, and the species in which these genes occur (Baquero 2012). Second, the index was analyzed for common modal patterns (i.e., principal components) across a diverse set of marine and freshwater ecosystems. Third, we sought to integrate the index into a public health surveillance framework in order to provide an example by which high-throughput metagenomic data can be applied to regulation or management.

## Methods

*Data sources*. Sequence reads for the 25 metagenomic samples included in this analysis are publicly available and were downloaded from the National Center for Biotechnology Information (NCBI) Sequence Read Archive (SRA; http://www.ncbi.nlm.nih.gov/sra). These 25 samples were divided into seven ecosystems: estuary, coastal ocean, freshwater lake, marina, river sediment, wastewater treatment plant (WWTP) effluent, and WWTP sludge (Table 1). The estuary data set includes surface water samples taken offshore in the northern (samples P1, P26) and central (P5, P28, P32) basins of Puget Sound, State of Washington (Port et al. 2012). Sampling site P26 was specifically located adjacent to the northern basin in the Strait of Juan De Fuca, State of Washington. The marina sample is from the central basin of Puget Sound and was taken near shore inside an urban marina and close to a source of freshwater input (Port et al. 2012). The coastal ocean samples were collected as part of an annual California Cooperative Oceanic Fisheries Investigations cruise in the Southern California Bight (Allen et al. 2012). Samples at seven stations were taken along hydrographic and nutrient gradients in near (samples GS257, GS263, GS264) and offshore (GS258, GS259, GS260, GS262) upwelling regions within the California Current Ecosystem. The term marine refers here to the estuary, marina, and coastal ocean samples. The freshwater lake sample is from a reservoir encompassing 59 square miles near Atlanta, Georgia, that serves as a drinking water supply for the city and is used for recreational activities (Oh et al. 2011). The river sediment samples were taken at intervals downstream from a WWTP discharge site in Patancheru, Hyderabad, India, that processes water from approximately 90 drug manufacturers (Kristiansson et al. 2011). The wastewater effluent, taken from a WWTP that discharges into Puget Sound, has an average daily inflow of 133 million gallons and is sourced from storm water/groundwater (53%), residential (29%), commercial (17%), and industrial (1%) processes (Port et al. 2012). The WWTP from which the activated sludge sample was obtained discharges into a local waterway in Charlotte, North Carolina, and has a daily inflow of 7.5 million gallons from primarily domestic sources in addition to several industries, a university, and a hospital (Sanapareddy et al. 2009).

**Table 1:** Metagenomic samples included in this study with associated metadata and summary statistics.

Characteristic	Estuary: Puget Sound, USA	Coastal ocean: California Bight, USA	Freshwater: Atlanta, GA, USA	Marina: Puget Sound, USA	River sediment: Patancheru, India	WWTP effluent: Seattle, WA, USA	WWTP sludge: Charlotte, NC, USA
No. of samples	5	12	1	1	4	1	1
Size fraction (μm)	0.2–3	0.1–0.8, 0.8–3	0.22–1.6	0.2–3	NA	0.2–3	NA
Depth (m)	5	2	5	1	0^*a*^	NA	NA
Megabase pairs	413	1,940	502	91	91	48	95
Mean read length (bp)	368	551	395	379	365	381	250
ORFs [(mean ± SD) %]	81.8 ± 2.1	69.4 ± 9.95	86.2	87.3	74.9 ± 0.565	89.7	89.8
Pfams [(mean ± SD) %]	40.8 ± 1.5	32.8 ± 8.9	39.8	45.3	30.5 ± 1.1	39.8	35.6
SRA accession no.	SRP015952	SRP006681	SRA023414	SRP015952	SRP002078	SRX328700	SRA001012
Reference	Port et al. 2012	Allen et al. 2012	Oh et al. 2011	Port et al. 2012	Kristiansson et al. 2011	Port et al. 2012	Sanapareddy et al. 2009
Abbreviations: NA, not applicable; ORF, open reading frame; Pfam, protein family. ^*a*^River sediment samples were collected at centimeters in depth along the shoreline of the river.							

All samples analyzed in this study (except the river sediment sample) were filtered and size fractionated (0.1–3.0 μm) to target the microbial community. Genomic DNA was extracted and shotgun sequenced using pyrosequencing (Margulies et al. 2005). Pyrosequencing of total genomic DNA was performed using 454 GS-FLX or GS-FLX Titanium technologies (454 Life Sciences, Branford, CT). For data sets with multiple samples (e.g., estuary, coastal ocean, river sediment), samples were individually barcoded and sequenced in parallel. Summary sequencing statistics, including functional annotation, are provided in Table 1. Open reading frames (ORFs) were predicted with MetaGeneMark software (http://topaz.gatech.edu/metagenome/) (Zhu et al. 2010) and protein domains assigned using the Pfam 26.0 database (Punta et al. 2012).

**Figure 1:**
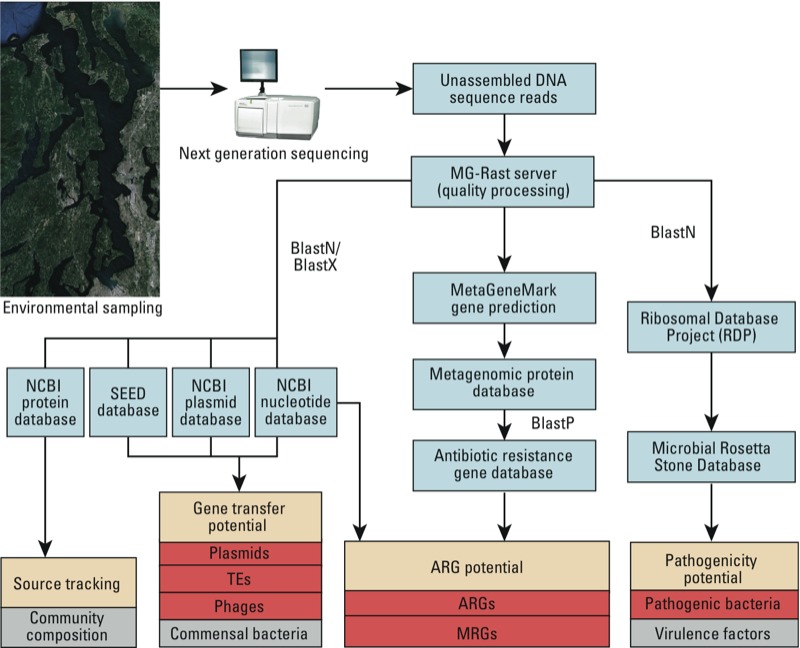
Bioinformatic framework for quantifying the index of ARDs. The index categories are shown in the cream-colored boxes and the subcategories in red. The gray boxes (e.g., commensal bacteria and virulence factors) represent subcategories that have not yet been incorporated into the index but may still play an important role in determining ARD potential. NCBI, National Center for Biotechnology Information.

*ARD index*. Metagenomic data relevant to environmental surveillance of ARDs was classified into three categories: gene transfer potential, ARG potential, and pathogenicity potential (Figure 1). A fourth category, source tracking, relates to identifying potential anthropogenic sources of ARDs through community composition profiling but has not yet been incorporated into the index analysis. The index categories were quantified via their respective subcategories as shown in Figure 1.

*Bioinformatic analyses*. The unassembled DNA sequence reads for each metagenome were run through a bioinformatic framework that quantified the ARD index (Figure 1). Reads were quality processed using the MG-Rast pipeline (Meyer et al. 2008) and then run through three separate analyses (one for each index category, excluding source tracking). Quality control parameters included the removal of reads that had a length > 2 SDs from the mean sample read length, > 5 ambiguous bases, < 5% of any one nucleotide, or 100% identity to another sequence over the first 50 bp.

The abundance of each index subcategory was calculated using different sequence similarity thresholds in order to generate a distribution of values for each subcategory and to determine how these thresholds affect data interpretation (Table 2). The high threshold represents the most conservative annotation approach (with the least false-positives), followed by a gradual reduction in stringency, including medium-high, medium-low, and low thresholds. Unless stated otherwise, annotated reads (per subcategory) were normalized to the total number of sequence reads per sample.

**Table 2:** Sequence similarity thresholds used to quantify the index subcategories.

Index category/subcategory	High	Medium-high	Medium-low	Low
Gene transfer potential
Plasmids	95% ID; 400 bp	95% ID; 300 bp	95% ID; 200 bp	95% ID; 100 bp
TEs	80% ID; 120 aa	80% ID; 90 aa	80% ID; 60 aa	80% ID; 30 aa
Phages	50% ID; 150 aa	50% ID; 100 aa	50% ID; 75 aa	50% ID; 50 aa
ARG potential
ARGs	80% ID; 150 aa	80% ID; 100 aa	80% ID; 75 aa	80% ID; 50 aa
MRGs	50% ID; 150 aa	50% ID; 100 aa	50% ID; 75 aa	50% ID; 50 aa
Pathogenicity potential
Pathogens	95% ID; 400 bp or 150 aa	95% ID; 300 bp or 100 aa	95% ID; 200 bp or 75 aa	95% ID; 100 bp or 50 aa
Abbreviations: aa, amino acids; bp, base pairs; ID, identity.				

Gene transfer potential subcategories included plasmids, TEs, and phages. Plasmids were annotated by BLASTN (http://blast.ncbi.nlm.nih.gov/Blast.cgi) searching (Altschul et al. 1990) the reads against plasmid sequences available in the NCBI RefSeq database (http://www.ncbi.nlm.nih.gov/refseq) (1,843 sequences) using the sequence similarity thresholds shown in Table 2. To identify TEs, 431,000 sequences annotated as TEs were downloaded from GenBank (http://www.ncbi.nlm.nih.gov/genbank/) and databased, and metagenomic reads were then searched against this database using the similarity thresholds. To annotate phages, reads were taxonomically assigned through the MG-Rast server using BLASTP (http://blast.ncbi.nlm.nih.gov/Blast.cgi), and reads matching to phage families or genera were retained for each similarity threshold. The total phage count for each metagenome was normalized to the total number of sequences assigned at the domain level.

ARG potential subcategories included ARGs and MRGs. ARGs were identified using the same approach as previously described (Port et al. 2012). Briefly, we compiled an ARG database (11,498 sequences) composed of a nonredundant and updated version of the Antibiotic Resistance Genes Database (http://ardb.cbcb.umd.edu/) (Liu and Pop 2009) in addition to ARGs from metagenomic samples that were functionally verified to confer resistance (Schmieder and Edwards 2012). Proteins were predicted from the ORFs generated from MetaGeneMark (Zhu et al. 2010) and then BLASTP searched against the ARG database (E-value < 10^–5^) using the thresholds presented in Table 2 to determine the best match. Sequences with similarity to MRGs were identified by searching the SEED database subsystem “Resistance to antibiotics and toxic compounds” (Overbeek et al. 2005). This subsystem contains genes and gene clusters encoding resistance to arsenic, mercury, and cadmium.

Two approaches were used to identify pathogenic bacteria.

Sequences were searched against the Ribosomal Database Project (Cole et al. 2009) at the similarity thresholds and species level; matches were then annotated as pathogens if present in the Microbial Rosetta Stone Database (Ecker et al. 2005). This database contains a list of bacterial pathogens known to pose a human health risk.
Sequences were taxonomically annotated using the lowest common ancestor algorithm (LCA) within the MG-Rast server (Meyer et al. 2008), and reads matching to the species level at each similarity threshold were retained and run against the Microbial Rosetta Stone Database (Ecker et al. 2005).

*Statistical analyses*. For principal component analysis (PCA), the abundance counts for each index subcategory were normalized to the total number of sequences in the index for a given sample. PCA was performed on the normalized data using the JMP version 10.0 statistical package (SAS Institute Inc., Cary, NC). Eigen vectors and loading values were extracted for the first two principal components. Finer scale analysis of the genomic elements composing each index subcategory was run using GraphPad Prism, version 6.0 (GraphPad Software, San Diego, CA). Abundance counts per genomic element were normalized to the total number of sequences within the respective subcategory and 95% confidence intervals were generated for each proportion.

## Results

**Figure 2:**
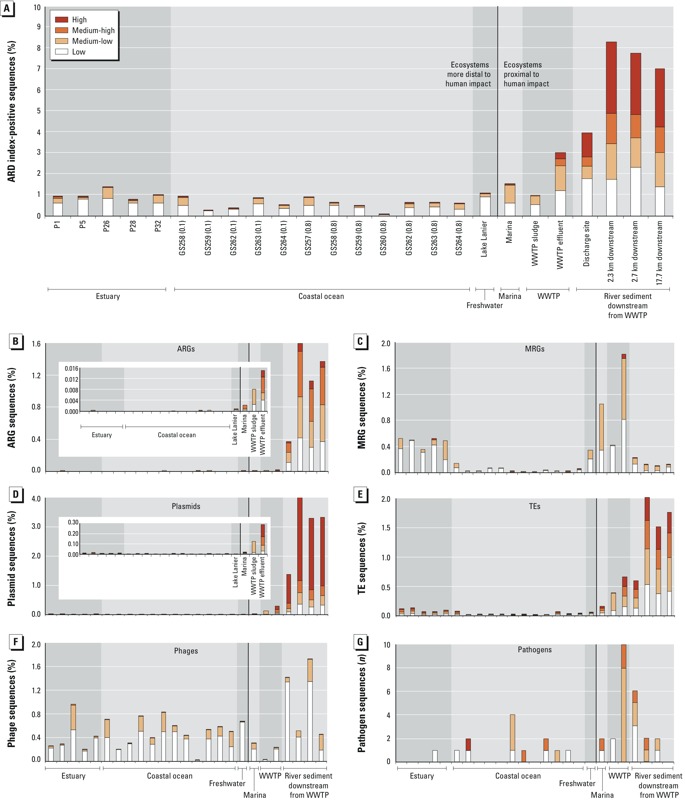
Percentage of total sequenced reads per metagenome assigned to the ARD index. (*A*) Percentage of index-positive sequences per sample and ecosystem and (*B–G*) the percentage of sequence reads per sample and ecosystem assigned to each index subcategory [(*B*) ARG sequences; (*C*) MRG sequences; (*D*) plasmid sequences; (*E*) TE sequences; (*F*) phage sequences; (*G*) pathogen sequences]. The percentages are shown for four different sequence similarity thresholds [including high, medium-high, medium, and low stringencies (see Table 2)]. The number of pathogen-annotated sequences is shown instead of the percentage. The vertical bar in each plot separates ecosystems more distal versus more proximal to human impact. Filter sizes (i.e., 0.1 and 0.8 μm) are listed after the station names for the coastal ocean samples. The graph inserts for ARGs and plasmids in *B* and *D* are zoomed-in views of the abundance of each subcategory excluding the river sediment samples.

*Antibiotic resistance potential*. An ARD index was developed that consisted of three categories related to the molecular etiology of antibiotic resistance: *a*) gene transfer, *b*) ARG, and *c*) pathogenicity potential. To first compare the antibiotic resistance potential across the samples, index scores were calculated for each metagenome using four sequence similarity thresholds ranging from high to low stringency (Table 2). When different bioinformatic thresholds are applied, the index scores change and consequently reveal differences that can impact public health monitoring and decision making. Application of the highest threshold generated the lowest percentage of index-positive sequences (mean, 0.025%) for all samples except the river sediment (Figure 2A). As the similarity thresholds are reduced, this percentage increases to 0.033% (medium-high), 0.28% (medium-low), and 0.55% (low). Individual index subcategories were also differentially sensitive to increases in alignment length and, therefore, threshold selection (Figure 2B–2G).

As hypothesized, environments most proximal to human impact had the highest cumulative ARD index scores at all similarity thresholds (Figure 2). Only the activated sludge sample did not follow this trend, likely because the average read length of the sludge data set did not meet the alignment length criteria of the higher thresholds. The river sediment samples taken downstream from a WWTP processing high volumes of antibiotics, as well as the effluent sample, had higher proportions of index-positive sequences due to elevated ARGs, plasmids, and TEs relative to the other samples (Figure 2B–2G; see also Supplemental Material, Table S1). The most impacted environments also had the largest proportion of sequences meeting the high similarity threshold. In particular, sequences from the river sediment data sets had strong matches to known plasmids. The estuary samples, on average, had a slightly increased cumulative score relative to the coastal ocean samples, with higher levels of MRGs, and to a lesser extent TEs, than the other marine samples. Sample P26 (estuary) had an elevated index score relative to all other marine samples due to an increased phage count (*Podoviridae*). Pathogens were rare at the higher similarity thresholds yet still detected in the effluent, river sediment, coastal ocean, and marina samples (Figure 2G; see also Supplemental Material, Table S1).

Multivariate analysis of all samples revealed ARGs and plasmids to be the most strongly correlated index subcategories at all sequence similarity thresholds (*r* = 0.85–0.98, *p* < 0.0001) (see Supplemental Material, Table S2).

*ARD index patterns*. We used PCA to identify modalities (or principal component “patterns”) for the metagenomic data associated with each sample. PCA reduces our highly multidimensional data set by generating weighted (or loaded) linear combinations [i.e., principal components (PCs)] of the metagenomic categories (e.g., ARGs, MRGs). As a result, a small number of PCs explain as much of the variance in the data set as possible. We ran PCA at the index subcategory level using the medium-high sequence similarity threshold for this case. For the abundance of genomic elements composing the index subcategories refer to Supplemental Material, Table S2 and Figure S1. In our analysis of the full set of samples, the first two principal components, PC1 and PC2, explained 68% of the total variance in the data set.

**Figure 3:**
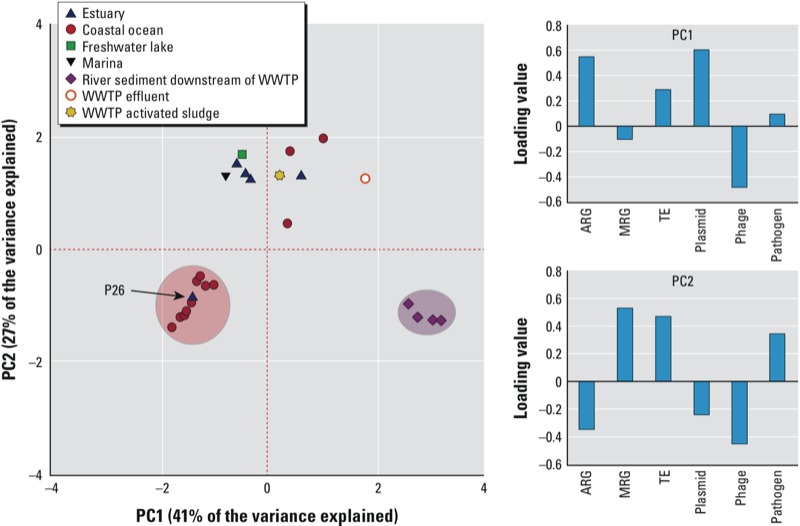
PCA score plot and corresponding loading values for the index subcategories by ecosystem. The medium-high sequence similarity threshold was used for this analysis (see Table 2). Sampling location P26 experiences increased mixing of oceanic waters relative to the other estuary samples. The red and purple circles indicate distinct coastal ocean and river sediment sample clusters, respectively, according to PC1.

PC1 was predominantly characterized by the presence (reflected by positive loadings) of ARGs, plasmids, and TEs and the relative absence (negative loadings) of phages, whereas PC2 reflected the presence of MRGs, TEs, and pathogens and the relative absence of ARGs, plasmids, and phages (Figure 3). There was a clear division between the coastal ocean and river sediment samples along PC1, whereas the estuary, freshwater lake, and WWTP effluent formed a mixed cluster with neutral scores along PC1. Despite the diversity of sample types and relatively small sample size, the marine locations were still largely distinguished from one another along PC2. The estuary samples had positive scores within PC2, whereas the coastal ocean samples were negative. The PC scores for the estuary samples are consistent with the presence of MRGs (arsenic and mercury resistance) and TEs (mainly *Rhodobacteraceae* sp.) and the relative absence of ARGs and plasmids, whereas the coastal ocean samples were characterized by phages (primarily *Myoviridae* and *Podoviridae*) and the relative absence of MRGs, TEs, and pathogens. The freshwater lake sample had a similar profile to the estuary, including the presence of MRGs (mainly arsenic resistance) and TEs (mainly *Ralstonia*, *Rickettsia,* and *Synechococcus* spp.). The PC results characterized the river sediment samples by the presence of ARGs (sulfonamide and aminoglycoside resistance genes) and plasmids (*Edwardsiella tarda* plasmid pEIB202, *Escherichia coli* pO26, and *Pasteurella multocida* plasmid pCCK38) and by the relative absence of MRGs (Figure 3; see also Supplemental Material, Table S2). The WWTP effluent was characterized by the presence of MRGs (arsenic and mercury resistance), pathogens *(Acinetobacter calcoaceticus*) and to lesser extent TEs, and the relative absence of phages. The effluent and activated sludge share positive scores in PC2. However, the sludge was nearly unweighted in PC1, whereas effluent was highly positive on that axis mainly because of an absence of phages.

PCA outliers included estuary sample P26 and three coastal ocean samples (GS259.1, GS260.8, and GS262.1). Site P26 experiences increased mixing of oceanic and Puget Sound waters relative to the other estuary locations, which may explain why it is grouped within the coastal ocean cluster. GS259.1 and GS262.1 are the only coastal ocean samples representing the 0.1-μm microbial community from oligotrophic waters, but there is no apparent relationship between distance offshore and the ARD index profile.

## Discussion

We developed and tested an index for characterizing the ARD potential of marine and freshwater environments using shotgun metagenomics. Currently available metagenomic data sets allow for gene transfer potential, ARG potential, and pathogenicity potential to be included in the index, although future introduction of source tracking data will enrich the approach. The index comprises an ecological context for ARD potential by providing both the prevalence of ARGs and the potential mechanisms by, and species in which, these genes may be passed. This index differed across both diverse environmental samples and also within a group of marine samples. Ecosystems proximal to human impact, including effluent and river sediment collected downstream from a WWTP processing high volumes of pharmaceuticals, had the highest cumulative index scores. These samples were distinguished by higher potentials for gene transfer, pathogenicity, and the presence of ARGs. Less impacted environments, including marine samples and a freshwater lake, had indices reflecting reduced public health concern but exhibited a distinct fingerprint characterized by either phages or MRGs, depending on location. Pathogens were rare across all data sets but were likely underestimated given the shotgun approach and, therefore, limited sequencing depth.

As the samples in this study were diverse, multiple factors may have contributed to the index profiles obtained including microbial community composition, ecosystem type, sampling methods, seasonality, and underlying data quality. We did not directly address community composition or seasonality, but composition is likely reflected in the ecosystem type. We aimed to minimize the impact of sample collection in part by only including studies that targeted the same size fraction. The PCA results suggest that ecosystem type is a stronger predictor of the index profiles than sequence quality. The coastal ocean samples had a wide range in the number of predicted ORFs and proteins, yet they clustered closely in the PCA score plots. Furthermore, the river sediment samples, which had a low number of ORFs and predicted proteins compared to the other samples (except coastal ocean), had the highest number of predicted ARGs and MGEs. Thus, although data quality may impact quantification of the index, the diverse nature of the samples confounded other potential factors. As more metagenomic data with greater spatiotemporal resolution become available, we will be better able to tease apart these factors.

We evaluated the choice of sequence similarity thresholds for annotating metagenomic data. Specific public health decisions may require the selection of different thresholds in order to optimize the balance of false-positives to false-negatives. Our sequence similarity thresholds matched or exceeded the criteria used in other studies investigating ARG and gene transfer in water. (Kristiansson et al. 2011; Zhang et al. 2011). There was a significant decrease in the number of index-positive sequences for each sample and index subcategory as the threshold was increased. This trend may be related to sequence read length in that sequences assigned at the lower thresholds may be too short to reach the alignment length criteria of the higher thresholds (e.g., WWTP sludge sample) or that the lower thresholds overassign false-positives. Further optimization of sequence similarity thresholds for public health applications will be necessary to ensure proper interpretation of the index.

*Applications to public health surveillance*. Current water quality standards are culture based and highly specific for targeted organisms. For example, beach and shellfishery closures in Washington State occur when fecal coliform levels exceed a geometric mean of 14 colony forming units (CFUs) or enterococci levels exceed a geometric mean of 70 CFU/100 mL marine water (State of Washington 2014). Although this has been an effective approach for reducing exposure to well-known pathogens, early risk management may benefit from population-level screening that results in a lower false-negative rate and thus increased sensitivity for a broader range of organisms or genes of interest. Furthermore, a reduction in specificity, and subsequent increase in false-positives, may not be appropriate for regulatory contexts, but it may be accepted when using a metric such as the ARD index to gain a broader understanding of the antibiotic resistance potential of an environmental sample and to detect the emergence of ARDs.

To frame metagenomic screening data within an early risk management approach, we can calculate an environmental detection rate for the ARD index by sample (see Supplemental Material, Figure S2). The environmental detection rate provides a rough estimate of the number of ARD sequences present per volume of water sampled (and takes into account the mass of DNA extracted), the mass sequenced, and sequencing depth. For example, the environmental detection rates for ARGs in the WWTP effluent and estuary samples were approximately 1.58 × 10^8^ sequences/L and 0 sequences/L, respectively (based on the medium-high sequence similarity threshold). Quantitative microbial risk assessments have used gene abundance counts (i.e., genome copies/L detected via quantitative polymerase chain reaction) for pathogenic markers in fecally contaminated recreational waters to determine pathogen dose (Staley et al. 2012). The environmental detection rate described above begins to lay out a similar approach for metagenomic assessments that may be informative for distinguishing differently impacted environments and evaluating a variety of public health impacts across marine microbial communities.

*Relevance to public health management*. Water quality management decisions have ignored ARDs or antibiotics, likely because of a lack of data and uncertainty regarding risk and risk metrics. Given the global magnitude of antibiotic resistance, including the emergence of multi-drug resistance bacterial strains in the environment, information pertaining to the status, patterns, and trends in ARDs is needed. Public health management decisions that may benefit from information regarding ARD potential include actions aimed at reducing the sources and exposure routes of ARDs and the framing of adaptive monitoring protocols. Source control of ARDs entering coastal environments primarily involves waste management and the regulation of antibiotic use in agriculture, aquaculture, hospitals, and households (Davies and Davies 2010). Exposure control of ARDs may involve beach or shellfish bed advisories or aquaculture siting. Due to the uncertainty in links between exposure and actual human health risk, current applications of the index as a screening tool are best suited to ARD source control. For example, using the index to screen WWTP and cruise ship effluent and discharge sites, freshwater inputs such as river mouths and coastal aquaculture operations could provide baseline environmental levels for anthropogenically sourced ARDs. The availability of such data would benefit decisions that currently do not account for the potential risk associated with antibiotic resistance release, such as reducing ARD dissemination into the environment by improving WWTP technologies or reducing the use of activated sludge as fertilizer for agricultural crops.

*Future data needs*. The ARD index is a high-throughput measure of ARD potential and as such cannot be directly related to human health risk. For environmentally sourced ARGs to pose a health risk, they must *a*) be transferable via MGEs; *b*) be transferred to either pathogenic or commensal bacteria that then infect or colonize humans; and *c*) confer resistance to antibiotics of clinical importance. Furthermore, vectors such as phages are ubiquitous in the marine environment (Breitbart 2012); thus any link to the dissemination of ARGs will require more targeted investigations. These limitations reflect the fact that, although emerging technologies will continue to provide unlimited access to genomic information, development of risk assessment frameworks will be of equal importance.

Although the cost and time required for metagenomic analysis is still greater than existing regulatory options for monitoring, advances in sequencing technologies and bioinformatic platforms are increasing the utility of high-throughput approaches. Next generation sequencing platforms now offer increased sequencing depths and read lengths for < $0.10/megabase (Glenn 2011). Furthermore, the availability of publicly available bioinformatic analysis tools and pipelines (Scholz et al. 2012) provides a platform for public health practitioners to access and automate in a way that addresses the research or regulatory question at hand.

Decreased sequencing costs and increased sequencing depths will also allow for longitudinal sampling and greater geospatial coverage, leading to a more comprehensive profiling of the ARD index. Furthermore, although the sample size in this study was limited, the PCA framework presented provides a platform from which to tease apart the index and characterize individual ecosystems.

## Conclusions

We had three objectives, to *a*) develop a metagenomic ARD index that quantifies the antibiotic resistance signal within marine and freshwater environments, *b*) analyze this index for common patterns characterizing specific ecosystems, and *c*) conceptually frame the index within an environmental health surveillance context. Significant differences were seen in the index when comparing marine and freshwater environments that differ in proximity to human impact, and distinct index patterns were evident across these environments. We conclude that the index has potential to be a valuable screening tool for early risk management of ARDs, but to define index threshold levels of concern and link these levels to decisions will require a better understanding of the prevalence, fate, and transport of ARGs in the marine environment. Nevertheless, characterization of the ARD potential of environmental microbial communities is a first step toward incorporating metagenomic information into monitoring frameworks for antibiotic resistance in aquatic ecosystems.

## Supplemental Material

(467 KB) PDF
