# Supplementary material for: Metagenomic Frameworks for Monitoring Antibiotic Resistance in Aquatic Environments
Source: Environ Health Perspect. 2013 Dec 13;122(3):222–8. doi: 10.1289/ehp.1307009 (PMC3948035; doi:10.1289/ehp.1307009)
Supplement: (467 KB) PDF [file ehp.1307009.s001.pdf]

Environmental Health Perspectives doi:10.1289/ehp.1307009

Supplemental Material
Metagenomic Frameworks for Monitoring Antibiotic Resistance in Aquatic Environments

Jesse A. Port, Alison C. Cullen, James C. Wallace, Marissa N. Smith, and Elaine M. Faustman

Table of Contents

Supplemental Material, Table S1. Most abundant genomic elements for each index subcategory by ecosystem..……………………………………………………………...…Page 2

Supplemental Material, Table S2. Pearson's correlation coefficients (r) for multivariate analysis of the index subcategories…………………………...………………………......Page 5

Supplemental Material, Figure S1. Relative abundance of the genomic elements composing the index subcategories by ecosystem…………….…………..…………………….…Page 6

Supplemental Material, Figure S2. Framework for estimating the environmental detection rate of ARDs in metagenomic samples using next generation sequencing………………...Page 7

Supplemental Material, Table S1. Most abundant genomic elements (e.g., gene class, species, host species) for each of the index subcategories by ecosystem type. Count data and relative abundance (percent out of all genomic elements within an index subcategory) are provided for each sample (except for pathogens where only count data is shown). This data was generated using the medium-high sequence similarity threshold described in Table 2.

**Genomic element**	**Estuary**	**Coastal ocean**	**Freshwater lake**	**Marina**	**WWTP sludge**	**WWTP effluent**	**River sediment**
**Antibiotic resistance genes (class)**							
Aminoglycoside	0	0	0	0	0	3 (25.0%)	248 (22.5%)
Bacitracin	0	0	0	2 (50.0%)	0	0	0
Beta lactam	0	0	0	1 (25.0%)	0	2 (16.7%)	2 (0.181%)
Chloramphenicol	0	0	1 (50.0%)	0	0	0	0
Macrolide	0	0	0	0	0	3 (25.0%)	53 (4.8%)
Multidrug	0	1	0	1 (25.0%)	0	0	1 (0.091%)
Quinolone	0	0	0	0	0	0	1 (0.091%)
Sulfonamide	0	0	0	0	0	0	796 (72.1%)
Tetracycline	0	0	0	0	0	4 (33.3%)	0
Trimethoprin	0	0	0	0	0	0	2 (0.181%)
Other	0	0	1 (50.0%)	0	0	0	1 (0.091%)
**Metal resistance genes (class)**							
Arsenic resistance	146 (61.9%)	72 (75.0%)	32 (78.0%)	59 (43.7%)	0	55 (34.3%)	26 (59.1%)
Cadmium resistance	0	0	0	0	0	0	2 (4.5%)
Mercury resistance	90 (38.1%)	24 (25.0%)	9 (22.0%)	76 (56.3%)	1 (100%)	105 (65.6%)	16 (36.4%)
**Plasmids (host)**							
Aeromonas sp.	0	0	0	0	0	8 (4.5%)	282 (4.6%)
Acinetobacter spp.	1 (1.6%)	0	2 (20.0%)	0	0	3 (1.7%)	180 (3.0%)
Comamonas testosteroni	0	0	2 (20.0%)	0	0	7 (3.9%)	1 (0.02%)
Cupriavidus metallidurans	1 (1.6%)	2 (2.0%)	0	0	0	31 (17.3%)	116 (1.9%)
Edwardsiella tarda	0	0	0	0	0	0	2,020 (33.3%)
Escherichia coli	1 (1.6%)	1 (1.0%)	1 (10.0%)	0	0	4 (2.2%)	1,877 (30.9%)
Klebsiella pneumonia	0	1 (1.0%)	0	3 (20.0%)	0	3 (1.7%)	15 (0.25%)
Lactococcus sp.	0	0	0	0	0	22 (12.3%)	1 (0.016%)

Genomic element	Estuary	Coastal ocean	Freshwater lake	Marina	WWTP sludge	WWTP effluent	River sediment
Nitrosomonas eutropha	0	0	0	2 (13.3%)	0	1 (0.56%)	0
Paracoccus spp.	0	0	0	0	0	9 (5.0%)	4 (0.065%)
Pasteurella multocida	0	0	0	0	0	0	1,113 (18.3%)
Rhodobacter sphaeroides	3 (4.9%)	2 (2.0%)	0	0	0	3 (1.7%)	0
Ruegeria spp.	52 (85.2%)	55 (55.0%)	0	4 (26.7%)	0	0	0
Salmonella enterica	0	1 (1.0%)	0	0	0	1 (0.56%)	122 (2.0%)
Sphingomonas spp.	0	9 (9.0%)	1 (10.0%)	0	0	8 (4.5%)	4 (0.066%)
Vibrio spp.	0	12 (12.0%)	0	1 (6.7%)	0	0	1 (0.016%)
Other	3 (4.9%)	17 (17.0%)	4 (40.0%)	5 (33.3%)	0	79 (44.1%)	338 (5.6%)
**Transposable elements (host)**							
Acinetobacter sp.	2 (0.21%)	0	0	0	1 (3.0%)	33 (4.2%)	7 (1.5%)
Alteromonas sp.	0	6 (1.4%)	0	14 (4.0%)	0	0	0
Escherichia coli	2 (0.21%)	2 (0.47%)	0	0	0	6 (0.76%)	38 (8.4%)
Flavobacteria bacterium	9 (0.92%)	0	0	0	10 (30.3%)	2 (0.25%)	1 (0.22%)
Klebsiella pneumoniae	0	0	2 (0.80%)	2 (0.58%)	1 (3.0%)	14 (1.8%)	190 (41.9%)
Mycobacterium sp.	4 (0.41%)	0	0	2 (0.58%)	1 (3.0%)	43 (5.4%)	3 (0.66%)
Nitrosomonas sp.	0	0	1 (0.40%)	15 (4.3%)		11 (1.4%)	5 (1.1%)
Octadecabacter sp.	175 (17.9%)	38 (8.9%)	0	29 (8.4%)	0	0	0
Polaromonas sp.	2 (0.21%)	0	5 (2.0%)	18 (5.2%)	0	14 (1.8%)	0
Pseudoalteromonas sp.	11 (1.1%)	30 (7.0%)	0	8 (2.3%)	0	0	0
Pseudomonas sp.	0	5 (1.2%)	18 (7.2%)	17 (4.9%)	2 (6.1%)	42 (5.3%)	7 (1.5%)
Ralstonia sp.	0	0	33 (13.2%)	0	0	9 (1.1%)	13 (2.9%)
Rhodobacteraceae bacterium KLH11	181 (18.0%)	77 (18.0%)	0	17 (4.9%)	0	0	0
Rickettsia sp.	2 (0.21%)	0	31 (12.4%)	0	0	2 (0.25%)	0
Roseobacter sp.	101 (10.4%)	26 (6.1%)	0	2 (0.58%)	0	6 (0.76%)	0
Salmonella enterica	0	0	0	0	0	0	57 (12.6%)
Synechococcus sp.	0	1 (0.23%)	27 (10.8%)	7 (2.0%)	0	0	0
Thalassiobium sp.	120 (12.3%)	50 (11.7%)	0	10 (2.9%)	0	0	0
Uncultured bacterium	24 (2.5%)	19 (4.4%)	11 (4.4%)	6 (1.7%)	0	47 (5.9%)	9 (2.0%)
Other	342 (35.1%)	174 (40.7%)	121 (48.6%)	199 (57.5%)	18 (54.5%)	564 (71.1%)	123 (27.1%)

Genomic element	Estuary	Coastal ocean	Freshwater lake	Marina	WWTP sludge	WWTP effluent	River sediment
**Phages**							
Inoviridae	0	0	0	0	0	0	3 (50.0%)
Myoviridae	18 (24.0%)	42 (47.2%)	16 (69.6%)	11 (42.3%)	1 (33.3%)	3 (3.7%)	0
Podoviridae	46 (61.3%)	37 (41.6%)	1 (4.3%)	10 (38.5%)	0	11 (13.4%)	3 (50.0%)
Siphoviridae	11 (14.6%)	10 (11.2%)	6 (26.1%)	5 (19.2%)	2 (66.7%)	63 (76.8%)	0
Tectiviridae	0	0	0	0	0	5 (6.1%)	0
**Pathogens**							
Acinetobacter calcoaceticus	0	0	0	0	0	2	0
Acinetobacter lwoffii	0	0	0	0	0	0	1
Aeromonas caviae	0	0	0	1	0	0	0
Ralstonia pickettii	0	0	0	0	0	0	1
Salmonella enterica	0	1	0	0	0	0	0
Staphylococcus epidermidis	0	1	0	0	0	0	0
Yersinia pestis	0	1	0	0	0	0	0

Supplemental Material, Table S2. Pearson's correlation coefficients (r) for multivariate analysis of the index subcategories. The values for each sequence similarity threshold (low, medium-low, medium-high, high) are shown for each comparison.

Index subcategory	ARG	MRG	Plasmid	TE	Phage	Pathogen
ARG						
MRG	-0.36, -0.31, -0.16, ND					
Plasmid	0.98***,0.94***, 0.90***, 0.85***	-0.31, -0.34, -0.07, ND				
TE	0.48, -0.01, -0.78***, -0.71***	0.26, -0.07, -0.17, ND	0.58*,0.23, -0.87***, -0.77***			
Phage	-0.52*, -0.45, -0.36, -0.19	-0.57*, -0.47, -0.07, ND	-0.59*, -0.56*, -0.39, -0.17	-0.82***, -0.55*, -0.02, 0.11		
Pathogen	-0.07, -0.06, -0.09, -0.09	0.21, 0.09, 0.01, ND	0.01, 0.04, -0.06, -0.15	0.31, 0.35, -0.03, -0.15	-0.22, -0.13, -0.18, -0.09	

Abbreviations: ARGs, antibiotic resistance genes; MRGs, metal resistance genes; ND, non-detect; TE, transposable elements. *p<0.01. **p<0.001. ***p<0.0001.

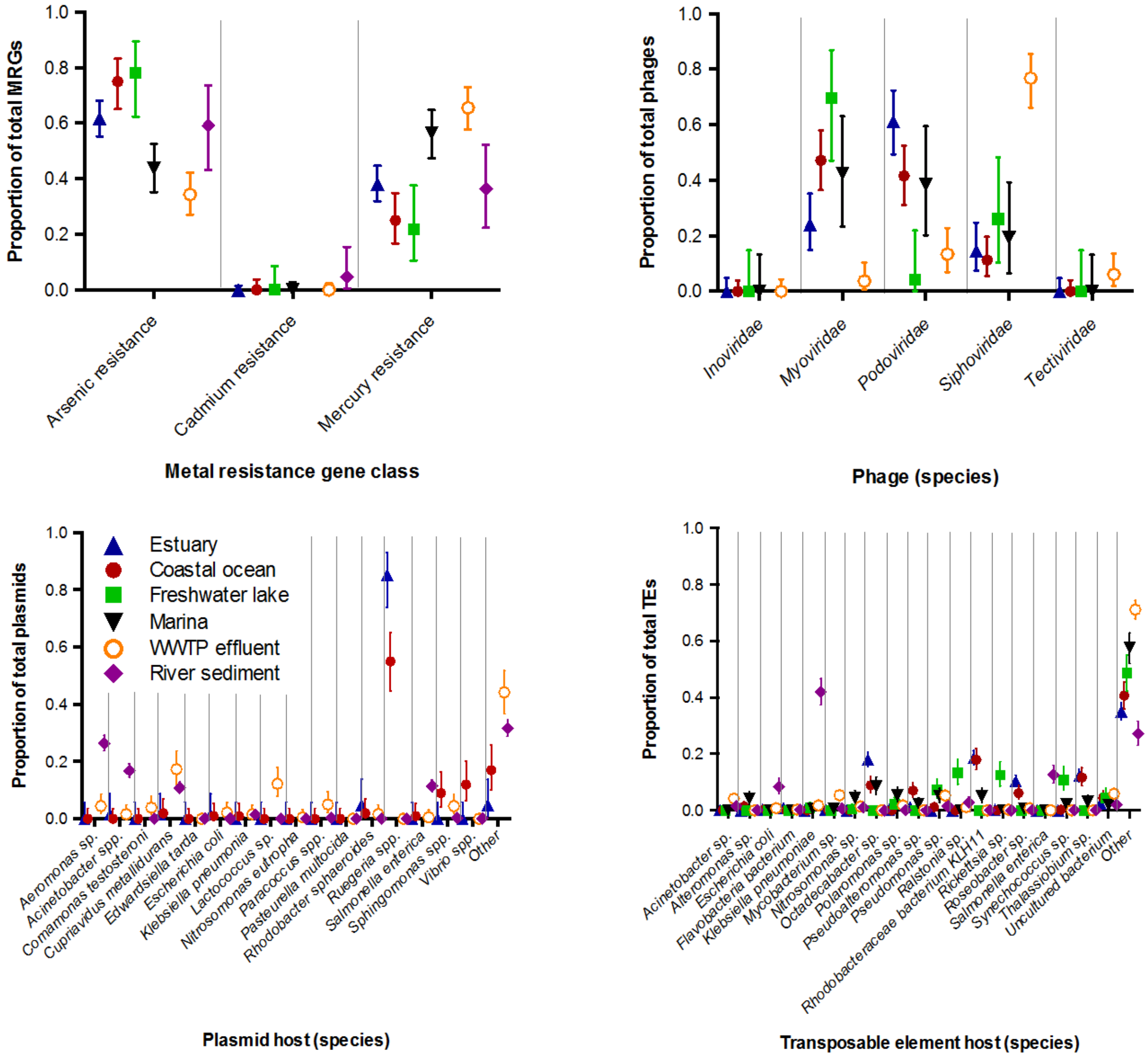

Supplemental Material, Figure S1. Relative abundance of the genomic elements composing the index subcategories. The number of sequences representing each genomic element in a given ecosystem was normalized to the total number of sequences for the respective index subcategory in that ecosystem. Bars represent 95% confidence intervals. A similar analysis was performed for antibiotic resistance genes (ARGs) and pathogens, but the sample size was sufficient to carry out the significance test for ARGs in the river sediment sample only. Ecosystems that are absent for a subcategory plot also had insufficient sample sizes. Abundances were generated using the medium-high sequence similarity threshold described in Table 2.

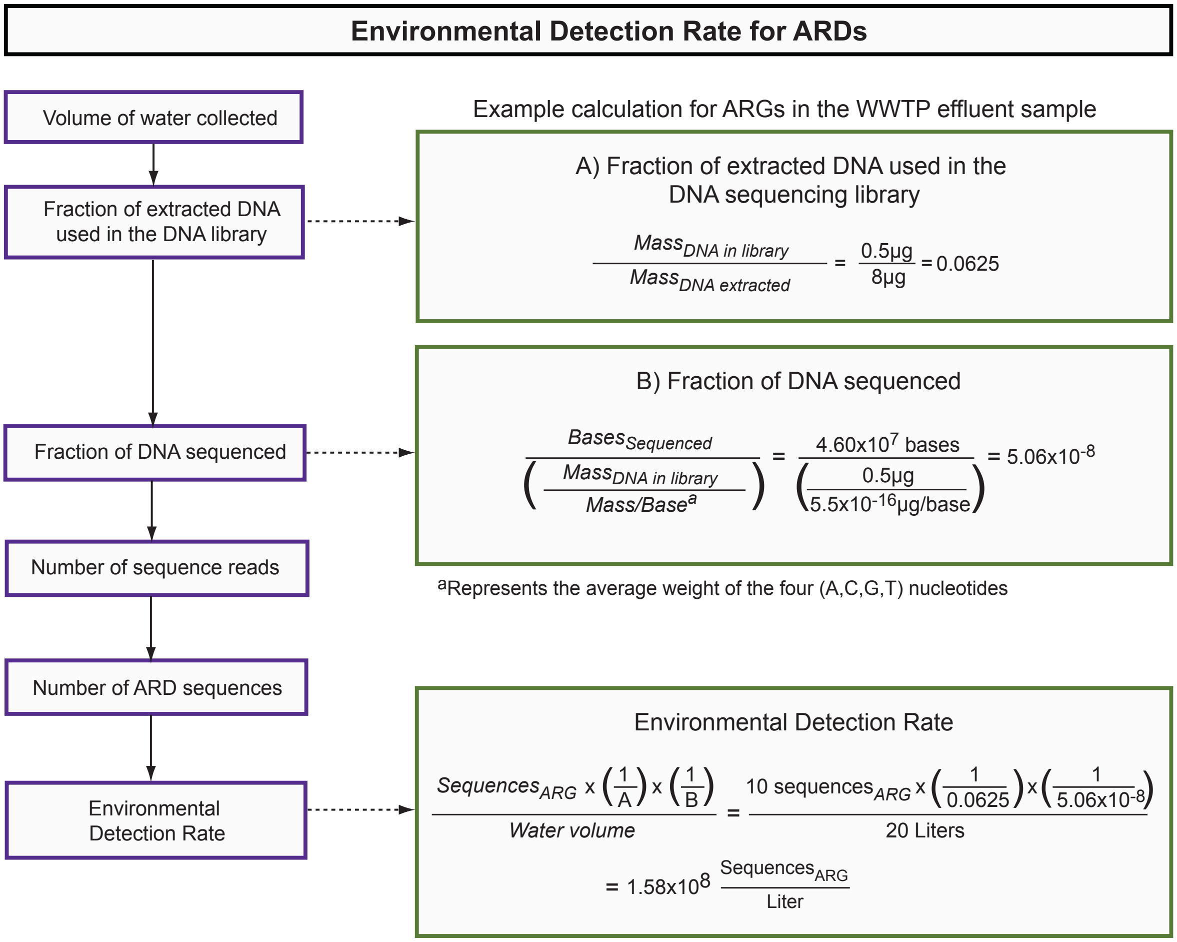

Supplementary Material, Figure S2. Mathematical framework for estimating the environmental detection rate of antibiotic resistance determinants (ARDs) in metagenomic samples using next generation sequencing. The environmental detection rate reflects the number of ARD-related sequences per liter of water filtered. An example calculation is shown for antibiotic resistance genes (ARGs) in the WWTP effluent sample (see Table 1 for description). The equations correct for the fraction of extracted DNA used as input for the DNA sequencing library (variable A) and the fraction sequenced (variable B).
